# Matching of XEN®45 and PRESERFLO™ MicroShunt cases: Outcomes of a 3-year follow-up

**DOI:** 10.1371/journal.pone.0335080

**Published:** 2025-10-31

**Authors:** Cemre Altas, Marcus Walckling, Tobias Brockmann, Fabian Stelzle, Anselm Jünemann, Thomas Fuchsluger

**Affiliations:** 1 Department of Ophthalmology, University Medical Center Rostock, Rostock, Germany; 2 Ernst-Abbe-Hochschule Jena, University of Applied Sciences, Jena, Germany,; 3 Ophthalmology Practice Stelzle, Burg, Germany; 4 Viselle Eye Centrum Erlangen, Erlangen, Germany; The University of Iowa, UNITED STATES OF AMERICA

## Abstract

**Objective:**

To compare long-term outcomes of XEN®45 (XEN) and PRESERFLO™ MicroShunt (PRESERFLO) implants in the surgical management of glaucoma over a 3-year follow-up period, focusing on intraocular pressure (IOP) reduction, need of medication, surgical success, and postoperative complications.

**Methods:**

A retrospective, matched case control study was conducted, including 42 eyes with preceded implantation of a XEN or PRESERFLO. Patients were statistically matched using SPSS algorithms for glaucoma diagnosis, previous surgery, and demographics. Standardized success criteria defined surgical outcomes: complete success (IOP ≤ 15 mmHg or ≤ 18 mmHg without medications) and qualified success (IOP ≤ 15 mmHg with < 3 medications). Postoperative care and needling or revision interventions were recorded. Statistical analyzes included Wilcoxon, Mann-Whitney U, and t-tests.

**Results:**

Both devices significantly reduced IOP and glaucoma medication over 3 years. XEN reduced IOP from 22.1 ± 8.6 mmHg to 13.5 ± 3.8 mmHg, while PRESERFLO reduced IOP from 22.1 ± 6.6 mmHg to 12.6 ± 4.0 mmHg (p > 0.05 for between-groups, 3 years postoperative). Medication use decreased from 2.4 ± 1.0 to 0.9 ± 1.4 for XEN and 3.1 ± 1.1 to 1.1 ± 0.9 for PRESERFLO (p > 0.05 for between-groups). Surgical success rates were similar after 3 years. PRESERFLO showed fewer postoperative revisions (28.6% vs. 42.9% for XEN; p > 0.05). Early hypotony occurred more frequently with XEN (17 vs. 7 cases; p = 0.029).

**Conclusion:**

Both XEN and PRESERFLO effectively reduce IOP and the need for glaucoma medication, with comparable surgical success rates after 3 years. PRESERFLO demonstrated a lower revision rate and reduced frequency of early postoperative hypotony. These findings support both implants as viable options for minimally invasive glaucoma surgery, with low risk profiles.

## Introduction

Glaucoma is a complex eye disease caused by several factors resulting in progressive optic nerve damage. As the disease progresses there is a loss of ganglion cells, a decrease in nerve fiber layer thickness and specific changes in the optic nerve associated with visual field loss [1–3]. In 2010, 2.1 million people worldwide were estimated as being legally blind due to glaucoma [4].

In addition to topical treatments, including eye drops, various surgical options are available [3,5,6]. Surgery can be the first choice or used if the optic nerve keeps deteriorating despite the topical treatment [7].

There is increasing interest in surgical implants for glaucoma treatment. The XEN45® stent (Allergan Inc., Dublin, Ireland) has been FDA-approved since November 2016 [8], while the PRESERFLO™ MicroShunt (Santen Pharmaceutical Co. Ltd., Osaka, Japan) received CE certification in Europe in 2012, with the commercial introduction in 2019 [9,10]. Both procedures share similar mechanisms of action, and initial studies report very good results with a low complication rate [9–15].

This study compared outcomes between these two minimally invasive bleb forming surgery (MIBS) implants in terms of intraocular pressure (IOP), number of pressure lowering medications, and follow-up procedures over a 3-year follow-up period in matched patient cases.

## Materials and methods

### Patient populations

In this retrospective, monocentric interventional case control study, 42 eyes each were selected after implantation of XEN^®^ 45 (XEN) and PRESERFLO^™^ MicroShunt (PRESERFLO). Matching was based on exact (or as statistically close as possible) matches for diagnosis, previous glaucoma surgery, and sex, with a ± 5-year tolerance for age. To avoid bias, matching was performed using automated ‘case-control matching algorithms in IBM SPSS Statistics (version 29.0.1.1 (244)) from a patient pool of 102 XEN cases (from 2014–2015) and 111 PRESERFLO cases (from 2019–2021). The indication for surgery and Inclusion criteria were established for patients showing progression of optic nerve damage or eye pressure values above the target pressure under maximum topical therapy, insufficient previous glaucoma surgery or eye drop intolerance.

Patients with uveitic secondary glaucoma, severely scarred conjunctiva following previous surgery, chronic blepharitis, conjunctivitis or a corneal thickness of more than 600 µm were excluded.

### Data collection

The study was approved by the Ethics Committee of the University Medical Center Rostock (Registration Number: A 2023−0002). The committee raised no ethical or professional objections to its implementation. Access to these data was granted solely for research purposes from 01/06/2024 to 31/08/2024. All personal information was anonymized, and it was ensured that no identifiable information of individual participants was used in the analysis. The data for this retrospective study were obtained through retrospective analyses of both paper and digital patient records (Filemaker™-based software “UAK-Data” until 10/2021 and Fidus Ophthalmology Software, Clinicianservice Wente GmbH, Darmstadt thereafter). Clinical examinations were performed according to a standardized protocol: Slit lamp-based anterior segment examinations including localization of the implant, relationship to the surrounding tissue, tissue alterations, bleb prominence and vascularization. The posterior segment, including optic nerve head and the retina, was examined using optical coherence tomography (OCT) for detailed imaging and fundoscopy for a broader visual assessment. The number of medications administered was determined either from previous records or by explicit questioning. Intraocular pressure was generally measured using Goldmann applanation tonometry except during the early postoperative period (< 2 weeks), when measurements were taken using rebound tonometry (iCare TA01i, iCare Finland Oy). The corneal thickness was measured by the Pentacam HR (OCULUS Optikgeräte GmbH, Wetzlar, Germany).

### Definition of success criteria

Surgical success was divided into three subgroups, with the first two groups (A-B) defining complete surgical success with varying degrees of severity and one group (C) representing qualified success. Complete surgical success was achieved with an IOP of: A: ≤ 15 mmHg and B: ≤ 18 mmHg - in each case without medication and without further pressure-lowering interventions. A qualified surgical success (C) corresponds to an IOP of ≤ 15 mmHg with < 3 topical IOP-lowering medications, without further pressure-lowering interventions. The success criteria were defined in accordance with the European Glaucoma Society’s ‘A Guide on Surgical Innovation for Glaucoma [16], with the exception that needling is considered equivalent to open bleb revision, as different filtering bleb management approaches were used for the two surgical procedures. Therefore, open bleb revision is not classified as a failure. However, if more than two bleb revisions are performed, this is considered a failure.

### Statistics

The statistical analysis was performed with IBM SPSS Statistics, version 29.0.1.1 (244). The number of medications was normally distributed (Kolmogorov-Smirnov test), so the test was performed using a t-test for paired or unpaired variables. The number of postoperative medications, six months and three years after surgery, revealed different variances between the two groups (Levene’s test, equality of variance), so that a Welch’s t-test was applied. IOP data did not show normal distribution (Kolmogorov-Smirnov test). Therefore, non-parametric tests such as the Wilcoxon test (testing of dependent variables) and Mann-Whitney U test (comparison between two independent variables) were used. Significance level was set at α = 0.05, Bonferroni correction was used for multiple testing.

### Material

The XEN (length: 6 mm, inner diameter 45 µm, outer diameter 150 µm) is composed of hydrophilic collagen and porcine gelatin. It provides a direct connection between the anterior chamber and the subconjunctival space. The material is solid in a dry environment but softens in a aqueous environment. The modification of the material is intended to reduce irritation and inflammatory reactions. Implantation is performed “ab interno” using a preloaded applicator that is inserted into the subconjunctival space of the superior nasal quadrant through the trabecular meshwork and sclera [17].

PRESERFLO (length: 8.5 mm, inner diameter: 70 µm, outer diameter: 350 µm) is made of poly(styrene-block-isobutylene-block-styrene) (SIBS). The implant creates an outflow of aqueous humor from the anterior chamber into the sub-tenon space. The implant is likewise used to drain the aqueous humor from the anterior chamber into the sub-tenon space. A small wing is used for fixation in the scleral pocket to prevent dislocation and leakage. Uniform outflow is intended to be achieved according to the Hagen-Poiseuille law of fluid mechanics [18].

### Intraoperative procedure

**XEN Procedure:** A 0.1 ml injection of mitomycin C 0.03% and 0.1 ml scandicain 2% was administered subconjunctivally and massaged into the nasal upper quadrant. The injection site, marked 3 mm from the limbus, was accessed via corneal paracentesis at 1 mm. After injecting viscoelastic into the anterior chamber, the needle was positioned in the chamber angle, pierced the eye wall, rotated to 12 o’clock, and the stent was deployed, leaving 1.5 mm visible in the anterior chamber. Ringer’s solution formed a uniform filtering bleb.

**PRESERFLO Procedure:** After placing a corneal traction suture at 12 o’clock, conjunctiva and Tenon’s capsule were opened over 4 clock-hours at the limbus and extended 8–10 mm to the equator. Mitomycin C (0.3 mg/ml) sponges were applied for 3 minutes and rinsed. A 1–2 mm scleral pocket was created 3 mm from the limbus, and a 25G cannula formed a channel into the anterior chamber. The MicroShunt was inserted, with its wings anchored in the scleral pocket, and the distal end positioned under Tenon’s pocket. The conjunctiva and Tenon’s were sutured to the limbus with vicryl or nylon 10−0.

### Postoperative treatment

During the hospitalization of the patient (~3 days after surgery), intraocular pressure was measured five times a day and a daily pressure profile was created.

**XEN Postoperative Care:** Topical therapy included preservative-free ofloxacin 0.3% (e.g., Floxal EDO®) five times daily for one week, dexamethasone 1.3% (e.g., Dexa EDO®) five times daily for four weeks (tapered monthly), and atropine 2% twice daily. After bleb needling, 1.25 mg/0.05 ml bevacizumab (Avastin®) was injected next to the filtering bleb for three consecutive days. Subconjunctival injections were not performed after the initial implantation.

**PRESERFLO Postoperative Care:** Topical therapy with preservative-free hypromellose 0.3% (e.g., Artelac EDO®), ofloxacin 0.3%, and dexamethasone 1.3% was applied five times daily for two weeks (tapered monthly). Bevacizumab (1.25 mg/0.05 ml, Avastin®) was injected next to the bleb for three consecutive days post-surgery and at one and two weeks postoperatively. Following bleb revisions, an additional 5 mg/0.1 ml 5-fluorouracil was administered.

Follow-up examinations were performed 1, 3, 6, 12, 24 and 36 months postoperatively included optical coherence tomography (SPECTRALIS™, Heidelberg Engineering) of the optic nerve head, nerve fiber density measurement, and 24−2 visual field with Swedish Interactive Testing Algorithm (SITA) Fast (Zeiss, Humphrey Field Analyzer 3™). Needling or open bleb revision was performed for elevated intraocular pressure (above target IOP)) or flat, scarred, or fibrocystic bleb configuration.

## Results

### Patient characteristics

42 eyes of 42 Caucasian patients, each selected after implantation of the XEN or PRESERFLO implantation.

The selection was made according to the following criteria

- **Diagnosis** (34 primary and 8 secondary open angle glaucoma pairs)
- **Previous pressure-lowering surgery** (1 pair filtering only, 14 pairs non-filtering only, 1 pair combined, total 16 pairs pre-surgery)
- **Sex** (22 female and 20 male)
- **Age ± 5 years** (XEN 69.6 ± 9.3 years, PRESERFLO 70.3 ± 9.3 years)

In 17 eyes (8 XEN vs. 9 PRESERFLO) one and in 17 eyes (9 XEN vs. 8 PRESERFLO) multiple preoperative IOP-lowering procedures were performed. The mean number of preoperative IOP-lowering procedures was 0.64 ± 0.90 (range: 1–4) – XEN 0.69 + 1.0 vs. PRESERFLO 0.60 ± 0.9 (p = 0.884). All case numbers for the respective follow-ups are shown in S1 Table.

### Intraocular pressure

**Fig 1:**
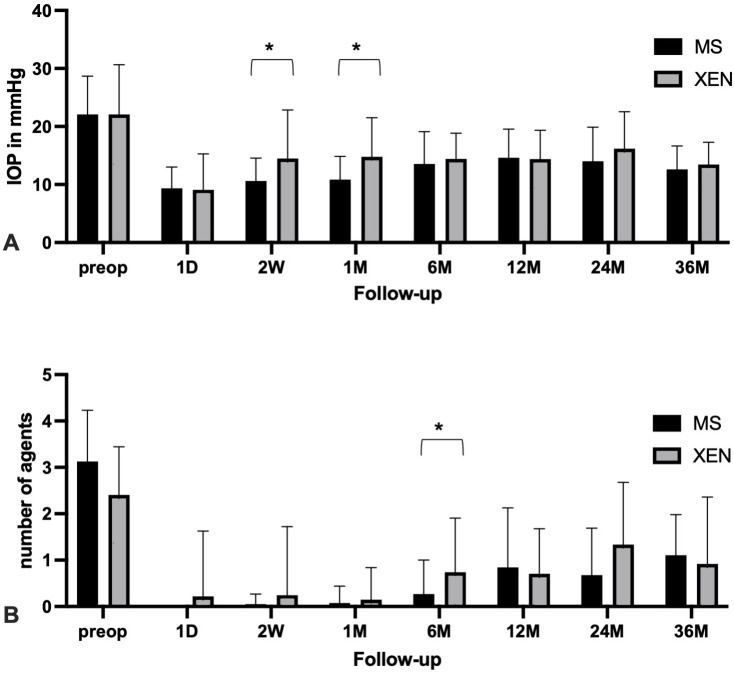
(A-B): Mean intraocular pressure (IOP) (A) and mean number of glaucoma medications (B) with standard deviations are shown for XEN (grey) and PRESERFLO (black) groups at baseline (preoperative), one day (1D), two weeks (2W), one month (1M) and 12, 24, and 36 months postoperatively. Asterisks (*) indicate statistically significant differences between groups at each time point (p < 0.05; Mann-Whitney U test for IOP, Welch’s t-test for medication).

In both groups, the mean IOP was significantly reduced over the entire 3-year follow up time (XEN p = 0.005; PRESERFLO p < 0.001; Wilcoxon test). XEN reduced the initial IOP from 22.1 ± 8.6 mmHg (n = 42) to 14.4 ± 5.0 mmHg (n = 35, 1 year), 16.2 ± 6.4 mmHg (n = 16, 2 years) and 13.5 ± 3.8 mmHg (n = 11, 3 years). Correspondingly, PRESERFLO reduced the initial IOP from 22.1 ± 6.6 mmHg (n = 42) to 14.6 ± 5.0 mmHg (n = 38, 1 year), 14.0 ± 5.9 mmHg (n = 30, 2 years) and 12.6 ± 4.0 mmHg (n = 19, 3 years). Only the 2-week and 1-month follow-up revealed a significant difference between the two methods (p = 0.023 and p = 0.008, respectively). There was no difference in IOP reduction between XEN and PRESERFLO. An overview of the IOP values over the entire course is summarized in Fig 1A.

### Medication

Both methods significantly reduced the number of glaucoma medications from 2.4 ± 1.0 to 0.9 ± 1.4, after 3 years (XEN, p = 0.017, paired t-test) and from 3.1 ± 1.1 to 1.1 ± 0.9 (PRESERFLO, p < 0.001, paired t-test), respectively. No significant differences between both procedures were observed (p = 0.444, Welch’s t-test). Except for the 6-month control, similar medication requirements were found for the implants (Fig 1B).

### Surgical success

At one year (XEN n = 35; PRESERFLO n = 37), 12 (34.2%) XEN and 17 (45.9%) PRESERFLO eyes achieved success category A (p = 0.346), 13 (37.1%) XEN vs. 22 (59.4%) PRESERFLO eyes achieved success category B (p = 0.065, Fishers exact test), and 17 (48.5%) XEN vs. 19 (51.4%) PRESERFLO eyes achieved success category C (p > 0.999).

After two years (XEN n = 16; PRESERFLO n = 30) the success rates in category A were: XEN 3 (18.8%) vs. PRESERFLO 15 (50.0%) eyes (p = 0.058), in category B: XEN 3 (18.8%) vs. PRESERFLO 17 (56.7%) eyes (p = 0.027) and in category C: XEN 7 (43.8%) vs. PRESERFLO 19 (63.3%) eyes (p = 0.229). The MicroShunt group demonstrated significantly better success rates in the category B.

After 3 years, the success rates were no longer different (XEN n = 11; PRESERFLO n = 19): category A: XEN 2 (18.2%) vs. PRESERFLO 4 (21.1%) eyes p (> 0.999), category B: XEN 2 (18.2%) vs. PRESERFLO 4 (21.1%) eyes (p > 0.999), category C: XEN 3 (27.3%) vs. PRESERFLO 10 (52.6%) eyes (p = 0.260).

### Revision surgery

Within the first year, 12 eyes (28.6%) underwent a single bleb revision by needling after XEN implantation, compared to 8 eyes (19.0%) requiring open revision after PRESERFLO, with no significant difference between the two methods (p = 0.442, Fisher’s exact test). Similar, no significant differences were observed after 2 or 3 years postoperative. After 2 years, the revision rates were 17 eyes (40.5%) following XEN and 11 eyes (26.2%) following PRESERFLO (p = 0.247). After 3 years, the rates were 18 eyes (42.9%) in the XEN group and 12 eyes (28.6%) in the PRESERFLO group (p = 0.255).

Multiple bleb revisions (two revisions) were performed in 7 eyes (16.7%) within 3 years after XEN and in 3 eyes (7.1%) after PRESERFLO (p = 0.313, Fisher’s exact test). Details for year 1 and 2 are provided in Table 1.

**Table 1:** Bleb revision and further IOP-lowering procedures.

	1 year			2 years			3 years		
	XEN	MS	p-value	XEN	MS	p-value	XEN	MS	p-value
Filtering bleb revisions (min. 1x)	12	8	0,442	17	11	0,247	18	12	0,255
Needling	12			16			17		
Open bleb revisions		8		1	11		1	12	
Filtering bleb revisions (max. 2x)	2	1	>0,999	4	2	0,676	7	3	0,313
pressure-lowering interventions	6	3	0,482	10	3	0,067	12	7	0,296
CPC	3	0		4	3		4	6	
Canaloplasty	1	0		1	0		1	0	
SLT	0	0		0	0		0	1	
iStent	0	0		1	0		2	0	
TE	1	0		3	1		4	1	
Revision/new implantation	1	0		1	0		2	0	
Explantation	0	0		0	1		0	1	
ALT, conjunctivoplasty	1	0		2	0		2	0	

MS – PRESERFLO MicroShunt, SLT – selective trabeculoplasty, CPC – cyclophotocoagulation, TE – trabeculectomy, ALT- argonlasertrabeculoplasty.

Following a single bleb revision, 11.8% of eyes in the XEN group and 18.2% in the PRESERFLO group achieved success category A after 2 years, while 5.6% of eyes in the XEN group achieved success category A after 3 years. None of the MicroShunt patients, who underwent a bleb revision within 3 years, achieved a categorized surgical success.

### Further IOP lowering procedures

Twelve eyes (28.5%) in the XEN group and 7 eyes (16.7%) in the PRESERFLO group required at least one additional IOP-lowering procedure within 3 years. Of these, 25.0% in the XEN group and 14.3% in the PRESERFLO group required multiple procedures (Table 1).

Follow-up procedures included cyclophotocoagulation (7.1% XEN; 14.3% PRESERFLO), TE (9.5% XEN, 2.4% PRESERFLO), canaloplasty (2.4% XEN), iStent (4.8% XEN), and selective laser trabeculoplasty (2.4% PRESERFLO).

Other procedures such as argon laser trabeculoplasty (2.4%) or conjunctivoplasty (2.4%) for bulbar hypotony were performed in the XEN group. In one eye of the PRESERFLO group, the flow of the tube could not be visualized during the open revision of the filtering bleb, so that an explantation with a subsequent TE was performed.

### Complications

In both groups, hypotony (IOP ≤ 5 mmHg) occurred in the early postoperative period; in 17 eyes (40%) after XEN, mainly immediately after surgery. In one case this maintained until the 1-month follow-up. After PRESERFLO, 7 eyes (17%) demonstrated transient hypotony early after surgery. Of these, 3 eyes showed clinically relevant hypotony up to 1 month and 1 eye showed fluctuating IOP between 5–9 mmHg (once 5 mmHg at the 6-month follow-up). Hypotony resolved in all eyes without further interventions by 3-month follow-up. Comparison of the two groups showed a higher rate of early postoperative hypotony in the XEN group, as compared to PRESERFLO (p = 0.0286, Fisher’s exact test).

In the early postoperative period, 2 (4.8%) eyes in both groups (p > 0.999) showed transient choroidal effusion. The longest duration (1 eye to 1 month postoperatively) was seen in the XEN group (no treatment required).

In the XEN group, we observed that the shunt dislocated into the anterior chamber in one eye and under the conjunctiva in another eye, requiring revision of the shunt. One eye had a Fuchs’ Delle requiring Tenon’s conjunctivoplasty. Another eye showed distortion of the shunt immediately postoperatively and a week later a fracture of the distal end without functional impairment, so that no repeated surgery was necessary. Similarly, one eye showed a kink in the shunt at the 1-month follow-up and continued to function well for up to 3 years.

## Discussion

This retrospective, matched case-control study compares the long-term outcomes of the XEN®45 Gel Stent and the PRESERFLO™ MicroShunt over a period of up to three years. Our analysis demonstrates that both procedures can achieve a significant and comparable reduction in intraocular pressure (IOP) and medication requirements, although differences in the temporal course and need for surgical revision were observed. Both devices have demonstrated good efficacy in previous studies: XEN in both combined procedure (cataract + XEN) and stand-alone [11–13,19], and the PRESERFLO with significant intraocular pressure reduction [9,10,14,15,20–23,24] and follow-up data for up to 5 years [14]. However, direct comparative studies between the two interventions remain limited. Our analysis shows that both procedures can achieve a significant and comparable reduction in IOP and medication requirements. Nonetheless, differences were observed in the temporal course and the need for surgical revision.

Our results demonstrate that both procedures (XEN and PRESERFLO) achieve a significant and sustained reduction in IOP over three years, with no significant differences in mid- and long-term IOP reduction. Only in the early postoperative phase (2 weeks and 1 month) did PRESERFLO show a significantly greater IOP decrease (p = 0.023 and p = 0.008, respectively), likely attributable to the ab externo implantation technique and more effective early bleb function. This finding is consistent with reports by Scheres et al. (2020) and Nobl et al. (2021), who also described superior early IOP control with PRESERFLO [25,26].

The reduction in medication requirements after three years was similarly effective in both groups (XEN from 2.4 to 0.9 agents; PRESERFLO from 3.1 to 1.1 agents), with no significant difference between the devices. These results align with existing literature that found no clear advantage of either implant regarding long-term medication reduction [25,26].

Regarding surgical success, using stricter criteria (Category A: IOP ≤ 15 mmHg without medication), PRESERFLO showed a trend toward higher success rates at one year (45.9% vs. 34.2% for XEN), though not statistically significant. After two years, a significant advantage for PRESERFLO emerged under Category B criteria (IOP ≤ 18 mmHg without medication; 56.7% vs. 18.8%, p = 0.027). These findings correspond with Wagner et al., who reported higher early success rates for PRESERFLO, and with Scheres et al., who observed comparable PRESERFLO success at two years [25,27]. Differences in patient selection and success criteria (e.g., management of bleb revision) complicate direct comparison with other studies [26,28].

Our lower success rates compared to Saletta et al., who reported complete success after one year in 62.2% (XEN) and 55.2% (PRESERFLO) of cases [28], are likely due to the substantially higher proportion of previously operated eyes (~40%) in our cohort, which negatively impacts prognosis.

A notable difference was observed in the rate of surgical revisions, with PRESERFLO showing a lower revision rate (28.6%) compared to XEN (42.9%), although this difference did not reach statistical significance. Our power analysis confirmed that the sample size (n = 42 per group) was sufficient to detect large effect sizes (Cohen’s d = 0.8) with high statistical power, whereas detecting moderate effects (e.g., d = 0.5) would have required a larger cohort (approximately 105 per group). Therefore, although no statistically significant difference was found, a moderate but clinically relevant difference cannot be excluded, and the possibility of a Type II error due to limited sample size must be acknowledged.

Mechanical complications, such as dislocation or fracture, occurred exclusively in the XEN group. Although similar events have also been described for PRESERFLO [15,20,21,29], and were also observed in our previous work [24], none of these complications occurred in this cohort. This difference may be attributed to the design and material properties of PRESERFLO, which is made of biocompatible SIBS and stabilized by anchoring fins, potentially offering greater mechanical stability [9]. Furthermore, the ab externo implantation technique with scleral pocket preparation likely enhances positional security and could reduce the risk of device migration. In contrast, XEN was implanted via an ab interno, closed-conjunctiva approach in our study, which may carry a higher risk for device movement or suboptimal positioning. Indeed, recent literature suggests that ab externo placement of XEN may offer improved control over stent positioning, reduce mechanical complications such as dislocation or occlusion [30].

The most common complication reported in the literature is transient hypotony (IOP ≤ 5 mmHg), which typically resolves spontaneously within one month. Contrary to our findings, some studies observed a higher incidence of hypotony following PRESERFLO implantation. Nobel et al. reported occasional prolonged hypotony events during follow-up, with intraocular pressure stabilizing spontaneously in 43% of cases, requiring conservative treatment (e.g., mydriatics or contact lenses) in 36%, or anterior chamber management with viscoelastic substances in 21% of MicroShunt patients. After XEN, spontaneous stabilization occurred in 56%, conservative therapy was needed in 31%, and anterior chamber revision with viscoelastic in 13% [26]. Scheres et al. found persistent hypotony in 8% of XEN eyes, whereas all PRESERFLO eyes reached IOP ≥ 6 mmHg within one month [25].

Higher rates of filtering bleb revision after XEN implantation (approximately 22–62%) have been documented [11,12,27,28,31]. One possible explanation is the difference in surgical techniques – ab interno for XEN versus ab externo for PRESERFLO. Interestingly, histological analyses by Neubauer et al. showed comparable levels of fibrosis in filtering blebs following XEN, PRESERFLO, and trabeculectomy, without evidence of increased lymphatic or granulocytic infiltration due to foreign-body reaction [32]. This suggests that the implant material itself does not trigger a stronger fibrotic or inflammatory response. Moreover postoperative management may also play a role; Nobl et al. reported a higher need for 5-fluorouracil after PRESERFLO than after XEN, potentially reflecting greater surgical trauma with PRESERFLO and a resulting lower tendency for scarring [26]. Conversely, the intraoperative use of a higher concentration of mitomycin C could also lead to less scarring, especially since the work of Beckers et al., Rabiolo et al. and Schlenker et al. has shown that this increases the efficacy of PRESERFLO, which in turn could be related to the configuration of the filtering bleb [21,33,34]. Furthermore in our study XEN eyes were more likely to undergo filtering bleb revision via needling. Theilig et al. demonstrated that open bleb revision was more effective after XEN than PRESERFLO [35,36], which could influence outcomes if both procedures were managed with open revision techniques.

A nother relevant factor for bleb configurationis the surgical technique employed. In our study, XEN was implanted using a closed conjunctival approach (ab interno). Currently, many centers favor the ab externo technique with an open conjunctiva to improve bleb morphology and longevity [30,37]. Indeed, the closed approach may contribute to the formation of avascular or insufficient blebs. However, no significant occurrence of avascular blebs was observed in our XEN cohort.

Our results at the respective time points align with previous studies, confirming good efficacy of both procedures after three years. The matched case-control design reduces selection bias and confounding, enhancing objectivity. However, several limitations merit discussion.

First, endothelial cell counts – a critical safety parameter, especially after concerns raised by devices like the CyPass – were not systematically recorded due to the retrospective design and lack of standardized measurements. This represents a relevant safety assessment gap that should be addressed in future prospective studies.

Second, although matching accounted for diagnosis, sex, age, and prior glaucoma surgeries, some potentially influential factors such as the interval since previous procedures and surgical technique details were not included. These unaccounted variables may have impacted postoperative wound healing and outcomes.

Third, follow-up at three years showed an imbalance, with fewer XEN eyes (n = 11) compared to PRESERFLO eyes (n = 19), introducing potential attrition bias. To evaluate whether this discrepancy was statistically significant, we conducted a Fisher’s Exact Test, which did not reveal a significant difference between the groups (p = 0.11). The earlier timing of XEN procedures in the observation period likely explains selective loss due to factors like relocation or referral patterns. While no evidence suggested bias from complications or failure, it cannot be ruled out.

Finally, although many centers may have transitioned from XEN®45 to XEN®63, our data remain relevant as XEN®63 was not available during the period of surgery (2014–2015), and long-term comparative data for XEN®63 versus PRESERFLO are still scarce. Thus, our findings provide valuable reference points for subconjunctival glaucoma implants.

Future research should address these limitations, by incorporating standardized endothelial cell assessments and evaluating the influence of surgical variables such as intraoperative mitomycin C concentration, postoperative antimetabolite use, and bleb management strategies. These factors, which differed between groups due to temporal and procedural variations, may influence outcomes and warrant further prospective studies for better differentiation between devices

## Conclusion

Both the XEN and the PRESERFLO are effective and safe procedures that provide significant pressure reduction for up to 3 years and reduce the need for pressure lowering medications. To date, there are minor differences that do not affect the overall clinical outcome.

## Supporting information

S1 Table Number of cases in each treatment groups: the table presents the number of cases in each treatment group – XEN and Preserflo (MS) – recorded over different postoperative time points. These time points include: preoperative (preop), 1 day (1D), 2 weeks (2W), 1 month (1M), 6 months (6M), 12 months (12M), 24 months (24M), and 36 months (36M). The case numbers indicate the sample size available for analysis at each follow-up interval.
(DOCX)
