# Supplementary material for: Matching of XEN®45 and PRESERFLO™ MicroShunt cases: Outcomes of a 3-year follow-up
Source: PLoS One. 2025 Oct 31;20(10):e0335080. doi: 10.1371/journal.pone.0335080 (PMC12578230; doi:10.1371/journal.pone.0335080)
Supplement: S1 Table — These time points include: preoperative (preop), 1 day (1D), 2 weeks (2W), 1 month (1M), 6 months (6M), 12 months (12M), 24 months (24M), and 36 months (36M). The case numbers indicate the sample size available for analysis at each follow-up interval. (DOCX) [file pone.0335080.s001.docx]

Supporting information

S1 Table

|  | preop | 1D | 2W | 1M | 6M | 12M | 24M | 36M |
| --- | --- | --- | --- | --- | --- | --- | --- | --- |
| XEN | 42 | 41 | 36 | 42 | 41 | 35 | 16 | 11 |
| MS | 42 | 42 | 40 | 38 | 36 | 37 | 30 | 19 |

Table 2: the table presents the number of cases in each treatment group – XEN and Preserflo (MS) – recorded over different postoperative time points. These time points include: preoperative (preop), 1 day (1D), 2 weeks (2W), 1 month (1M), 6 months (6M), 12 months (12M), 24 months (24M), and 36 months (36M). The case numbers indicate the sample size available for analysis at each follow-up interval.
